# ﻿Overview of hirsutella-like anamorphs in *Ophiocordyceps* (Sordariomycetes, Ophiocordycipitaceae): introducing two new species and one new record from China

**DOI:** 10.3897/mycokeys.119.145174

**Published:** 2025-07-01

**Authors:** Shi-Wen Xie, De-Ping Wei, Jun-Zhi Qiu, Xing-Can Peng, Ji-Chuan Kang, Zhang-Jiang He, Zeng-Zhi Li, Chun-Ru Li, Shi-Ke Huang, Xian Zhang, Zhong-Liang Liu, Jing Bu, Nalin N. Wijayawardene, Ting-Chi Wen

**Affiliations:** 1 School of Pharmacy, Guizhou University, Guiyang 550025, Guizhou, China; 2 State Key Laboratory of Green Pesticide; Key Laboratory of Green Pesticide and Agricultural Bioengineering, Ministry of Education, Guizhou University, Guiyang 550025, China; 3 Engineering Research Center of Southwest Bio-Pharmaceutical Resources, Ministry of Education, Guizhou University, Guiyang 550025, China; 4 State Key Laboratory of Agricultural and Forestry Biosecurity, College of Life Sciences, Fujian Agriculture and Forestry University, Fuzhou 350002, China; 5 Center of Excellence in Fungal Research, Mae Fah Luang University, Chiang Rai 57100, Thailand; 6 School of Science, Mae Fah Luang University, Chiang Rai 57100, Thailand; 7 Zhejiang BioAsia Life Science Institute, Pinghu 314200, China; 8 Center for Yunnan Plateau Biological Resources Protection and Utilization, College of Biology and Food Engineering, Qujing Normal University, Qujing 655011, China

**Keywords:** Entomopathogenic fungi, hirsutella-like, *Ophiocordyceps*

## Abstract

*Ophiocordyceps*, a species-rich genus in Ophiocordycipitaceae, is a holomorphic genus in which most of the species are reported with hirsutella-like anamorphs. In this study, we introduce two new species of hirsutella-like anamorphs from lepidopteran larvae (viz., *Ophiocordycepstielingensis***sp. nov.** and *Ophiocordycepskeqinii***sp. nov.**). *Ophiocordycepsradiata* (syn. *Hirsutellaradiata*), a new combination, exhibits a pathogenic association with a fly, and it is reported as a new geographic record from China, based on integrated morphological and molecular analyses. We provide a checklist of *Ophiocordyceps* species with hirsutella-like anamorphs and comprehensively review their characteristics of anamorphs and teleomorphs. These definitive findings establish a foundation for the classification and diversity of *Ophiocordyceps* species with hirsutella-like anamorphs.

## ﻿Introduction

The clavicipitoid fungi are an ecologically important group that are classified into Clavicipitaceae, Cordycipitaceae, Ophiocordycipitaceae, and Polycephalomycetaceae (Xiao et al. 2023). Members of these families establish close associations with insects (up to 13 orders of Insecta) and other arthropods (Wei et al. 2022). Ophiocordycipitaceae is a diverse family encompassing fungi with significant ecological, economic, medicinal, and cultural importance. Sung et al. (2007a) established Ophiocordycipitaceae based on molecular data, and this family currently comprises more than 500 species and eight genera, including *Drechmeria*, *Harposporium*, *Hantamomyces*, *Ophiocordyceps*, *Paraisaria*, *Purpureocillium*, *Tolypocladium*, and *Torrubiellomyces* (Quandt et al. 2014; Spatafora et al. 2015; Mongkolsamrit et al. 2019; Crous et al. 2020; Araújo et al. 2022). The type genus of Ophiocordycipitaceae, *Ophiocordyceps*, was erected by Petch (1931) to accommodate *O.blattae*, *O.unilateralis*, *O.peltata*, and *O.rhizoidea*. These four mentioned species share similarities in producing fibrous, tough, pliant to wiry, dark to brightly colored stromata; superficial to immersed perithecia; clavate asci with thickened apex; and whole, hyaline, fusiform, multiseptate ascospores. *Ophiocordyceps* is the most species-rich genus within Ophiocordycipitaceae, with a wide distribution ranging from tropical forests to temperate ecosystems. Anamorphs belonging to *Hirsutella*, *Hymenostilbe*, *Syngliocladium*, *Paraisaria*, and *Tilachlidiopsis* have been linked to species of *Ophiocordyceps* (Quandt et al. 2014; Mongkolsamrit et al. 2019).

*Hirsutella* is a widely distributed entomopathogenic genus with a broad host range, primarily infecting arthropods and nematodes (Liang 1990b). *Hirsutella* was originally classified as a clavarioid basidiomycete (Patouillard 1892). Speare (1920) re-evaluated the type species and clarified the taxonomic placement of this genus. Gams and Zare (2003) summed up that *Hirsutella* is distinguished by its basally subulate phialides, which taper into one (typically) or occasionally several very slender needle-like necks, either on synnemata or mononematous mycelium. Quandt et al. (2014) proposed that species with hirsutella-like anamorphs are phylogenetically spread throughout *Ophiocordyceps*, for which *Hirsutella* was suppressed in favor of *Ophiocordyceps*. However, there are still a few new species being introduced to this genus since then, viz., *H.tortricicola* (Zou et al. 2016a), *H.shennongjiaensis* (Zou et al. 2016b), *Hirsutellachangbeisanensis* (Qu et al. 2017), *H.hongheensis* (Yuan et al. 2020), *H.flava* (Qu et al. 2021), and *H.kuankuoshuiensis* (Qu et al. 2021). On the contrary, most researchers accepted the suggestion of Quandt et al. (2014) and added new species with hirsutella-like anamorph to *Ophiocordyceps*, such as *O.myrmicarum* (Simmons et al. 2015a), *O.nooreniae* (Crous et al. 2016), *O.retorta* (Qu et al. 2018), *O.unituberculata* (Wang et al. 2018), *O.sporangifera* (Xiao et al. 2019), *O.delicatula* (Clifton et al. 2021), *O.pingbianensis* (Chen et al. 2021), *O.flavida* (Mongkolsamrit et al. 2021), *O.nujiangensis* (Sun et al. 2022), *O.lilacina* (Mongkolsamrit et al. 2023), *O.maybankeae* (Tan et al. 2023), and *O.albastroma* (Sun et al. 2024).

During our field surveys of entomopathogenic fungi in southwestern China, we collected several samples of dead insects. In morphology, three fungal species were identified as hirsutella-like anamorphs. DNA sequence-based phylogenetic analyses confirmed two species (from lepidopteran larvae) are new to *Ophiocordycepss. str.* (viz., *O.tielingensis* sp. nov. and *O.keqinii* sp. nov.). *H.radiata* has been reclassified as *O.radiata* based on a newly collected specimen (associated with a fly), and it is the first time to report this species from China. Furthermore, a checklist of *Ophiocordyceps* species with hirsutella-like anamorphs and a comprehensive review of their teleomorphic and anamorphic characteristics are also provided.

## ﻿Methods and materials

### ﻿Sample collection and morphological study

A survey of entomopathogenic fungi was conducted in mixed forests in Yunnan and Liaoning Provinces of China. Two species were found infecting lepidopteran larvae, with their synnemata protruding from the host on the ground, while one species was found infecting flies attached to fresh fern leaves. High-resolution images and morphological data were collected in the field for subsequent taxonomic validation. The fresh samples were collected into sterilized self-sealing bags or centrifuge tubes and labeled appropriately. For a more detailed examination of the morphology of the specimens, freehand sections were made. Following sectioning, the tissue slices were carefully transferred onto slides using sterile water or Congo red solution for mounting. Subsequently, the prepared specimens were examined under a compound microscope (Nikon ECLIPSE Ni) to discern the intricate microstructures, including synnemata, phialides, and conidia. The dried specimens were deposited in the Herbarium of Cryptogams, Kunming Institute of Botany, Academia Sinica (KUN-HKAS). Index Fungorum identifiers were obtained following the protocols described in Index Fungorum (http://www.indexfungorum.org/, retrieved on 23 May 2025).

### ﻿DNA extraction, PCR amplification, and sequencing

Genomic DNA was extracted from fungal tissues using a DNA extraction kit (Omega Bio-Tek, Norcross, GA, USA) in accordance with the manufacturer’s protocol. The obtained total genomic DNA was stored at -20 °C. PCR amplification was performed for five loci, including the partial small subunit rRNA gene (SSU), the partial large subunit rRNA gene (LSU), the internal transcribed spacer encompassing the 5.8S rDNA gene (ITS), the translation elongation factor 1-alpha gene (*tef1-a*), and the partial RNA polymerase II largest subunit (*rpb1*). The corresponding primers that were used for the amplification and sequencing of these loci were NS1/NS4 for SSU (White et al. 1990), LROR/LR5 for LSU (Vilgalys and Hester 1990), ITS5/ITS4 for ITS (White et al. 1990), EF1-983F/EF1-2218R for *tef1-a* (Rehner and Buckley 2005), and CRPB1A/RPB1Cr for *RPB1* (Castlebury et al. 2004). The polymerase chain reaction (PCR) was performed in a 25 µL volume, including 12.5 µL of PCR mixture (2× Rapid Taq Master Mix, Vazyme Biotech), 7.5 µL of double-distilled water, 1 µL of each primer (10 µM), and 3 µL of 30 ng/µL DNA template. Amplifications of ITS, SSU, and LSU genes were carried out using a BioRAD T100 Thermal Cycler (Singapore) with the PCR program as follows: initial denaturation at 95 °C for 5 min, followed by 40 cycles of denaturation at 95 °C for 30 s, annealing at 55 °C for 50 s, extension at 72 °C for 30 s, and a final extension at 72 °C for 10 min. The PCR conditions of *tef1-a* and *rpb1* were as follows: 95 °C for 5 min, followed by 10 cycles of 95 °C for 30 s, 56 °C for 50 s, 72 °C for 50 s, 30 cycles of 95 °C for 30 s, 52 °C for 50 s, and 72 °C for 50 s, and end with 72 °C for 10 min. The PCR products were sent to Sangon Biotech (Shanghai) Co., Ltd. in Chongqing, China, for sequencing using the aforementioned primers. The generated sequences were manually edited using BioEdit v.7.0.5.3 (Hall 1999) and submitted to GenBank. The accession numbers of newly generated sequences are listed in Table 1.

**Table 1.** GenBank accession numbers of the taxa used in the phylogenetic analyses; the newly generated sequences are in bold. Ex-type strains are indicated by ‘T.’

Species	Voucher	Host	ITS	SSU	LSU	*tef1-a*	*rpb1*	Reference
Hirsutellacf.haptospora	ARSEF 2228	Diptera	KM652166	KM652075	KM652118	KM652001	KM652041	Simmons et al. (2015b)
*H.changbeisanensis*	GZUIFR hir160527	Homoptera	KY415578			KY415592		Qu et al. (2017)
*H.citriformis*	ARSEF 1035	Hemiptera	KM652153	KM652064	KM652105	KM651989	KM652030	Simmons et al. (2015b)
*H.cryptosclerotium*	ARSEF 4517	Hemiptera	KM652157	KM652066	KM652109	KM651992	KM652032	Simmons et al. (2015b)
*H.eleutheratorum*	ARSEF 13375	Coleoptera		MH057734		MH057732	MH057733	Wraight et al. (2018)
*H.fusiformis*	ARSEF 5474	Coleoptera		KM652067	KM652110	KM651993	KM652033	Simmons et al. (2015b)
*H.gigantea*	ARSEF 30	Hymenoptera			JX566977	JX566980	KM652034	Simmons et al. (2015b)
*H.guyana*	ARSEF 878	Hemiptera	KM652158	KM652068	KM652111	KM651994	KM652035	Simmons et al. (2015b)
*H.haptospora*	ARSEF 2226	Acari	KM652159			KM651995	KM652036	Simmons et al. (2015b)
*H.hongheensis*	HKAS 102451^T^	Insecta	MN017176	MN017177	MN017175	MN733824		Yuan et al. (2020)
*H.illustris*	ARSEF 5539	Hemiptera	KM652160	KM652069	KM652112	KM651996	KM652037	Simmons et al. (2015b)
*H.kuankuoshuiensis*	GZUIFR 2012KKS3-1	Lepidoptera	KY415575		KY415582	KY415590	KY945360	Qu et al. (2017)
*H.leizhouensis*	GZUIFR hir130707	Lepidoptera	KY415573			KY415587	KY945358	Qu et al. (2017)
*H.liboensis*	ARSEF 9603	Lepidoptera	KM652163	KM652072	KM652115			Simmons et al. (2015b)
*H.necatrix*	ARSEF 5549	Acari	KM652164	KM652073	KM652116	KM651999	KM652039	Simmons et al. (2015b)
*H.nodulosa*	ARSEF 5473	Lepidoptera	KM652165	KM652074	KM652117	KM652000	KM652040	Simmons et al. (2015b)
‘*H.radiata*’	ARSEF 1369	Diptera		KM652076	KM652119	KM652002	KM652042	Simmons et al. (2015b)
*H.rhossiliensis*	ARSEF 2931	Nematodes	KM652168	KM652078	KM652121	KM652004	KM652043	Simmons et al. (2015b)
*H.satumaensis*	ARSEF 996	Lepidoptera	KM652172	KM652082	KM652125	KM652008	KM652047	Simmons et al. (2015b)
*H.shennongjiaensis*	GZUIFR Snj121022^T^	Dermaptera			KY945357		KY945364	Qu et al. (2021)
*H.sinensis*	ARSEF 6282	Lepidoptera	KM652173	KM652083	KM652126	KM652009	KM652048	Simmons et al. (2015b)
*H.strigosa*	ARSEF 2197	Hemiptera	KM652175	KM652085	KM652129	KM652012	KM652050	Simmons et al. (2015b)
*H.subulata*	ARSEF 2227	Lepidoptera	KM652176	KM652086	KM652130	KM652013	KM652051	Simmons et al. (2015b)
*H.thompsonii*	ARSEF 3323	Acari	KM652188	KM652096	KM652143	KM652024	KM652059	Simmons et al. (2015b)
*H.thompsonii*	ARSEF 253	Acari	KM652179	KM652088	KM652133	KM652016		Simmons et al. (2015b)
*H.uncinata*	MTCC 10896	Myrtales	KJ524691		KJ524712			Seifert and Boulay (2004)
*H.versicolor*	ARSEF 1037	Hemiptera		KM652102	KM652150	KM652029	KM652063	Simmons et al. (2015b)
*Ophiocordycepsacicularis*	OSC 128580	Coleoptera	JN049820	DQ522543	DQ518757	DQ522326	DQ522371	Sung et al. (2007a)
*O.acroasca*	YFCC 9016^T^	Formicinae		ON555841	ON555922	ON567761	ON568681	Tang et al. (2023c)
*O.ansiformis*	YHH 2210007	Formicinae		OR345230		OR098435	OR351952	Tang et al. (2023b)
*O.arborescens*	NBRC 105891^T^	Lepidoptera	AB968398	AB968386	AB968414	AB968572		Ban et al. (2015)
*O.australis*	HUA 186097	Hymenoptera		KC610786	KC610765	KC610735	KF658662	Sanjuan et al. (2015)
*O.basiasca*	YHH 20191	Formicinae		ON555828	ON555910	ON567748	ON568672	Tang et al. (2023c)
*O.bifertilis*	YFCC 9012^T^	Formicinae		ON555843	ON555923	ON567763	ON568143	Tang et al. (2023c)
*O.blattae*	BCC 34765	Blattodea				MT533484	MT533478	Mongkolsamrit et al. (2020)
*O.blattae*	BCC 38241	Blattodea			MT512657	MT533485	MT533479	Sung et al. (2007a)
*O.borealis*	MFLU 18-0163	Coleoptera	MK863251	MK863044	MK863051	MK860189		Zha et al. (2021)
*O.campes*	BCC 36938^T^	Lepidoptera	MT783955		MT118175	MT118167	MT118183	Tasanathai et al. (2020)
*O.camponoti-atricipis*	ATRI3	Hymenoptera		KX713666	KX520652	KX713677		Araújo et al. (2018)
*O.camponoti-balzani*	G143	Hymenoptera		KX713658	KX713595	KX713690	KX713705	Evans et al. (2011)
*O.camponoti-bispinosi*	OBIS4	Hymenoptera		KX713637	KX713615	KX713692	KX713720	Araújo et al. (2015)
*O.camponoti-femorati*	FEMO2	Formicidae		KX713663	KX713590	KX713678	KX713702	Araújo et al. (2018)
*O.camponoti-hippocrepidis*	HIPPOC	Formicidae		KX713655	KX713597	KX713673	KX713707	Araújo et al. (2018)
*O.camponoti-nidulantis*	NIDUL2	Formicidae		KX713640	KX713611	KX713669	KX713717	Araújo et al. (2018)
*O.camponoti-rufipedis*	G108	Hymenoptera		KX713659	KX713594	KX713679	KX713704	Evans et al. (2011)
*O.clavata*	NBRC 106961	Coleoptera	JN943327	JN941727	JN941414	AB968586	JN992461	Sung et al. (2007a)
*O.communis*	BCC 1842	Termitidae			MH753680	MK284266	MK214110	Tasanathai et al. (2019)
*O.communis*	BCC 1874	Termitidae			MH753679	MK284267	MK214109	Tasanathai et al. (2019)
*O.communis*	BCC 2754	Termitidae	MH754727		MH753681	MK284268	MK214111	Sung et al. (2007a)
*O.contiispora*	YFCC 9027^T^	Formicinae		ON555832	ON555913	ON567752	ON568142	Tang et al. (2023c)
*O.delicatula*	ARSEF 14442^T^	Hemiptera		MZ198251		MZ246828	MZ246829	Clifton et al. (2021)
*O.elongata*	OSC 110989	Lepidoptera			EF468808	EF468748	EF468856	Sung et al. (2007a)
*O.entomorrhiza*	KEW 53484	Insecta		EF468954	EF468809	EF468749		Sung et al. (2007a)
*O.flabellata*	YFCC 8795^T^	Formicinae		OL310721	OL310724	OL322688	OL322687	Tang et al. (2023a)
*O.flavida*	BCC 84256^T^	Hemiptera			MT512655	MT533482	MT533476	Mongkolsamrit et al. (2021)
*O.formosana*	TNM F13893	Tenebrionoidea		KJ878908		KJ878956	KJ878988	Wang et al. (2015)
*O.fusiformis*	BCC 93025 ^T^	Termitidae	MZ676743		MZ675422	MZ707849	MZ707855	Tasanathai et al. (2022)
*O.geometridicola*	BCC 79823	Lepidoptera			MF614648	MF614632	MF614663	Luangsa-ard et al. (2018)
*O.globiceps*	MFLUCC 18-0495^T^	Diptera	MH725815	MH725811	NG068274	MH727387		Xiao et al. (2019)
*O.globiperitheciat*a	HKAS 126130^T^	Termitidae	OR015963	OR082950	OR015968	OR030532	OR119834	Fan et al. (2024)
*O.globosa*	BCC 93023	Termitidae	MZ676740		MZ675419	MZ707846	MZ707861	Tasanathai et al. (2022)
*O.hydrangea*	YFCC 8834^T^	Hemiptera		OM304635	OM304639	OM831276	OM831279	Zou et al. (2022)
*O.isopterae*	MY 12376.01	Termitidae	MZ676741		MZ675420	MZ707847	MZ707859	Tasanathai et al. (2022)
*O.issidarum*	MFLU 17-0751^T^	Hemiptera	NR160481		NG064454			Hyde et al. (2017)
***O.keqinii***	**HKAS 135614** ^T^	**Lepidoptera**	**PP951447**	**PP958849**	**PP956623**	**PP966946**		**This study**
*O.khokpasiensis*	BCC 48071	Termitidae	MH754728		MH753682	MK284269	MK214112	Tasanathai et al. (2019)
*O.khonkaenensis*	BCC 81463	Hemiptera	MK632044	MK632127	MK632102	MK632076	MK632169	Crous et al. (2019)
*O.kimflemingiae*	SC09B	Formicinae		KX713631	KX713620	KX713698	KX713724	Araújo et al. (2018)
*O.kobayasii*	BCC 75694^T^	Gryllidae	MK632030	MK632112	MK632082	MK632056	MK632172	Thanakitpipattana et al. (2020)
*O.krachonicola*	BCC 79667	Gryllidae	MK632047		MK632081	MK632055	MK632162	Thanakitpipattana et al. (2020)
*O.lanpingensis*	YHOS0707^T^	Lepidoptera		KC417459	KC417461	KC417463	KC417465	Chen et al. (2013)
*O.laojunshanensi*s	HKAS 126041	Lepidoptera	OQ935368		OP962379	OQ440732		Chen et al. (2011)
*O.laotii*	BCC 76495^T^	Formicidae	ON763786		ON764219	ON759347	ON759354	Mongkolsamrit et al. (2023)
*O.liangshanensis*	YFCC 15099244	Hepialidae			OQ608804	OQ622100	OQ622106	Wang et al. (2021b)
*O.lloydii*	OSC 151913	Formicidae		KJ878924	KJ878891	KJ878970	KJ879004	Sung et al. (2007a)
*O.longissima*	NBRC 108989	Odonata	AB968407		AB968421	AB968585		Sung et al. (2007a)
*O.longistipes*	HKAS 126186	Termitidae	OR015960	OR082947	OR015966	OR030531	OR062225	Fan et al. (2024)
*O.longistromata*	BCC 4449^7^T	Lepidoptera	MT783956		MT118178	MT118170		Tasanathai et al. (2020)
*O.macroacicularis*	NBRC 105888	Lepidoptera	AB968401	AB968389	AB968417	AB968575		Ban et al. (2015)
*O.maybankeae*	BRIP 72909b^T^	Coccinellidae	OR750694		OR731501	OR737805		Tan et al. (2023)
*O.mosingtoensis*	BCC 30904	Termitidae	MH754732		MH753686	MK284273	MK214115	Tasanathai et al. (2019)
*O.multiperitheciata*	BCC 69008	Lepidoptera			MF614657	MF614641		Luangsa-ard et al. (2018)
*O.myrmecophila*	BCC 82255	Hymenoptera	MH028143		MH028156	MH028183	MH028168	Sung et al. (2007a)
*O.nooreniae*	BRIP 5536^3^T	Termitidae		KX673811	KX673810	KX673812		Crous et al. (2016)
*O.nuozhaduensis*	YHH 20168	Formicinae		ON555849	ON555927	ON567769	ON568683	Tang et al. (2023c)
*O.ootakii*	J13	Formicinae		KX713652	KX713600	KX713681	KX713708	Araújo et al. (2018)
*O.ovatospora*	YHH 2206001^T^	Termitidae	OP295105	OP295110	OP295113	OP313801	OP313803	Tang et al. (2022)
*O.pauciovoperitheciata*	BCC 45562	Lepidoptera			MF614651	MF614634	MF614666	Luangsa-ard et al. (2018)
*O.pingbianensis*	YFCC 807^5^T	Coleoptera	MT273118		MT270099	MT270097	MT270098	Chen et al. (2021)
*O.pseudoacicularis*	BCC 53843	Lepidoptera			MF614646	MF614630	MF614661	Luangsa-ard et al. (2018)
*O.pseudorhizoidea*	BCC 86431^T^	Termitidae	MH754721		MH753674	MK284262	MK751469	Tasanathai et al. (2019)
*O.pseudorhizoidea*	NHJ 12522	Termitidae	JN049857	EF468970	EF468825	EF468764	EF468873	Tasanathai et al. (2019)
*O.pseudorhizoidea*	NHJ 12529	Termitidae		EF468969	EF468824	EF468765	EF468872	Tasanathai et al. (2019)
*O.purpureostromata*	TNS F18430	Coleoptera		KJ878931	KJ878897	KJ878977	KJ879011	Quandt et al. (2014)
***O.radiata***	**HKAS 135613**	**Diptera**		**PP958850**	**PP956622**			**This study**
*O.radiciformis*	BCC 93036	Termitidae	MZ676746		MZ675425	MZ707852	MZ707857	Tasanathai et al. (2022)
*O.ravenelii*	OSC 110995	Coleoptera		DQ522550	DQ518764	DQ522334	DQ522379	Sung et al. (2007a)
*O.salganeicola*	Mori01	Blattaria			MT741719	MT759575	MT759578	Araújo et al. (2021)
*O.salganeicola*	Mori02	Blattaria			MT741718	MT759572	MT759579	Araújo et al. (2021)
*O.satoi*	J7	Termitidae		KX713653	KX713599	KX713683	KX713711	Araújo et al. (2018)
*O.sinensis*	YHH 1805	Lepidoptera		MK984568	MK984580	MK984572	MK984587	Wang et al. (2022)
*O.sobolifera*	KEW 78842	Hemiptera		EF468972	EF468828		EF468875	Sung et al. (2007a)
*O.spataforae*	MY11765	Hemiptera/Coleoptera			MG831747	MG831746	MG831748	Luangsa-Ard et al. (2018)
*O.spataforae*	NHJ 12525	Hemiptera/Coleoptera		EF469125	EF469078	EF469063	EF469092	Sung et al. (2007a)
*O.spataforae*	OSC 128575	Hemiptera/Coleoptera	JN049845	EF469126	EF469079	EF469064	EF469093	Sung et al. (2007b)
*O.sphecocephala*	OSC 110998	Hymenoptera		DQ522551	DQ518765	DQ522336	DQ522381	Sung et al. (2007a)
*O.spicatus*	MFLU 18-0164	Coleoptera	MK863254	MK863047	MK863054	MK860192		Zha et al. (2021)
*O.stylophora*	OSC 111000	Coleoptera	JN049828	DQ522552	DQ518766	DQ522337	DQ522382	Sung et al. (2007a)
*O.subtiliphialida*	YFCC 8815^T^	Formicinae		ON555833	ON555914	ON567753	ON568673	Tang et al. (2023c)
*O.termiticola*	BCC 1770	Termitidae			MH753677	MK284264	MK214107	Tasanathai et al. (2019)
*O.termiticola*	BCC 1920	Termitidae			MH753678	MK284265	MK214108	Tasanathai et al. (2019)
*O.tricentri*	NBRC 106968	Hemiptera	AB968410	AB968393	AB968423	AB968593		Sung et al. (2007a)
***O.tielingensis***	**HKAS 135612^T^**	**Lepidoptera**	**PP951446**	**PP958848**	**PP956621**	**PP966945**	**PP955355**	**This study**
*O.unilateralis*	OSC 128574	Hymenoptera		DQ522554	DQ518768	DQ522339	DQ522385	Sung et al. (2007a)
*O.unituberculata*	YHH HU 1301^T^	Lepidoptera	KY923211	KY923213		KY923215	KY923217	Wang et al. (2018)
*O.variabilis*	ARSEF 5365	Dipteran		DQ522555	DQ518769	DQ522340	DQ522386	Sung et al. (2007a)
*O.xuefengensis*	GZUH2012HN14 ^T^	Coleoptera	KC631802	KC631789		KC631793	KC631798	Wen et al. (2013)
*Tolypocladiumcylindrosporum*	YFCC 1805001	Soil	MK984581	MK984565	MK984577	MK984569	MK984584	Wang et al. (2022)
*T.pseudoalbum*	YFCC 875^T^	Soil	OP207725	OP207717	OP207737	OP223151	OP223129	Dong et al. (2022)
*T.reniformisporum*	YFCC 1805002 T	Lepidoptera	MK984582	MK984566	MK984578	MK984570	MK984585	Wang et al. (2022)
*T.subparadoxum*	NBRC 106958	Soil	OP207727	OP207715	OP207735	OP223149	OP223127	Dong et al. (2022)
*T.yunnanense*	YFCC 878	Soil	OP207730	OP207720	OP207740	OP223154	OP223132	Dong et al. (2022)

Abbreviations: **ARSEF**: The Agricultural Research Service Collection of Entomopathogenic Fungi, USDA, USA; **BCC**: BIOTEC Culture Collection, Klong Luang, Thailand; **BRIP**: Queensland Plant Pathology Herbarium, Australia; **GZUH/GACP**: Herbarium of Guizhou University, China; **GZUIFR**: Institute of Fungal Resources of Guizhou University, China; **HKAS**: Kunming Institute of Botany, Academia Sinica, China; **HUA**: Herbarium Antioquia University, Medellin, COL; **KEW**: Mycology collection of Royal Botanical Garden, Surrey, UK; **MFLU**: Mae Fah Luang University Herbarium, Thailand; **MFLUCC**: Mae Fah Luang University Culture Collection, Chiang Rai, Thailand; **MTCC**: Microbial Type Culture Collection and Gene Bank, India; **MY**: Mycology Laboratory in BIOTEC, Thailand; **NBRC**: Biological Resource Center, the National Institute of Technology and Evaluation, Japan; **OSC**: Oregon State University Herbarium, Corvallis, Oregon, USA; **TNS F**: The mycological herbarium of the National Museum of Nature and Science, Tsukuba, Ibaraki, Japan; **YFCC**: Yunnan Fungal Culture Collection of Yunnan University, China; **YHH**: Yunnan Herbal Herbarium, China; **YHOL**: Yunnan Herbal Laboratory, Institute of Herb Biotic Resources, China; Holotype of specimens ATRI3/G143/OBIS4/FEMO2/HIPPOC/NIDUL2/G108/SC09B and J13 were deposited in **INPA** herbarium (Instituto Nacional de Pesquisas da Amazônia, Brazil).

### ﻿Phylogenetic analyses

The taxa included in the phylogenetic analyses were selected based on BLAST search results in NCBI and relevant literature (Quandt et al. 2014; Simmons et al. 2015b; Qu et al. 2021; Peng et al. 2024). Each locus was independently aligned using MAFFT version v.7 (Kuraku et al. 2013; Katoh et al. 2019). Uninformative gaps and ambiguous regions were removed using Trimal v.1.2 (Capella-Gutiérrez et al. 2009) with the -gt value set to 0.6. SequenceMatrix 1.7.8 (Vaidya et al. 2011) was used to combine the five trimmed alignments. AliView v. 1.26 (Larsson 2014) was used to convert the format to a FASTA file for maximum likelihood (ML) analysis and a NEXUS file for Bayesian inference (BI) analysis. The final combined alignment was used for ML and BI analysis.

ML analysis was performed using RAxML-HPC2 on ACCESS (8.2.12) (Stamatakis 2014) available in the CIPRES Science Gateway platform with the GTRCAT model and bootstrap iterations setting to 1000. The best-fit models for each gene were independently determined by MrModeltest version 2.3 (Nylander 2004) with Akaike Information Criterion (AIC), resulting in the selection of GTR+I+G for SSU, LSU, ITS, *tef1-a*, and *rpb1*. BI analysis was performed with MrBayes on XSEDE version 3.2.7a on the CIPRES Science Gateway portal, employing the suggested best-fit models and launching two parallel runs with four parallel Markov Chain Monte Carlo chains sampled every 1000 steps for 100,000,000 generations until the average standard deviation reached 0.01. The first 20% of trees represented burn-in fractions were discarded, and the remaining trees were used to calculate the posterior probabilities (PP) of each clade (Alfaro and Holder 2006). Phylograms generated from ML and BI analyses were viewed with the FigTree v.1.4.0 program (Rambaut and Drummond 2012) and edited with Adobe Illustrator.

## ﻿Results

### ﻿Phylogenetic analyses

**Figure 1.**
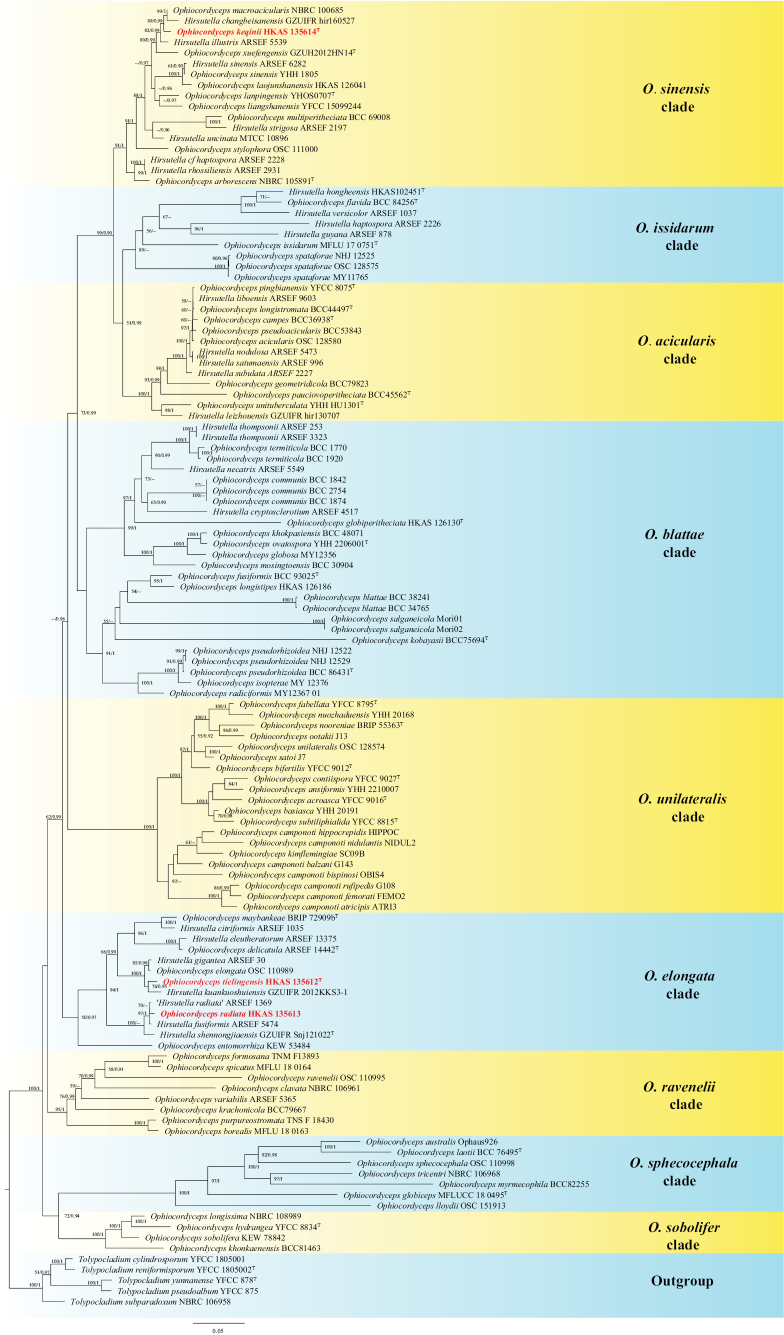
Phylogram generated from maximum likelihood analysis based on combined SSU, LSU, ITS, *tef1-a*, and *rpb1* sequence data. ML bootstrap values equal to or greater than 50% and PP values equal to or greater than 0.90 are given above each node. The newly generated sequences are indicated in red.

The combined dataset of 122 taxa consisted of 3959 characters (SSU: 1028 bp, LSU: 839 bp, ITS: 547 bp, *tef1-α*: 903 bp, and rpb1: 642 bp), of which 2238 characters were constant, 356 variable characters were parsimony-uninformative, and 1365 characters were parsimony-informative. Four strains of *Tolypocladium* were selected as the outgroup taxon. Both maximum likelihood (ML) and Bayesian inference (BI) analyses produced congruent tree topologies. The optimal ML tree with a likelihood score of -52,290.517614 (Fig. 1) resolved nine strongly supported clades, namely *O.sinensis*, *O.issidarum*, *O.acicularis*, *O.blattae*, *O.unilateralis*, *O.elongata*, *O.ravenelii*, *O.sphecocephala*, and *O.sobolifera*. *Ophiocordycepstielingensis* and *Hirsutellakuankuoshuiensis* formed a monophyletic group sister to *O.elongata* and *H.gigantea* (100% ML/1 PP; Fig. 1), nested within the *O.elongata* clade. *Ophiocordycepsradiata* (HKAS 135613) clustered with *H.radiata* and *H.fusiformis*, forming a clade sister to *H.shennongjiaensis* (100% ML; Fig. 1), also within the *O.elongata* clade. *Ophiocordycepskeqinii* was resolved as sister to a clade containing *O.macroacicularis* and *H.changbeisanensis* with moderate support (89% ML/0.99 PP; Fig. 1). The alignments used in this study are available on Figshare (https://doi.org/10.6084/m9.figshare.29075552).

### ﻿Taxonomy

#### ﻿ Ophiocordyceps tielingensis

Taxon classificationFungiSordariomycetesOphiocordycipitaceae

S. W. Xie, T. C. Wen & D. P Wei sp. nov.

9B95383D-2CA5-5CEB-BB50-790F544208C9

Index Fungorum: IF903218

Fig. 2

##### Etymology.

Named after the location where the type specimen was found, ‘Tieling’ County, Liaoning Province, China.

##### Description.

***Anamorph*: *Stromata*** extending from the body of a lepidopteran larva, simple, up to 70 mm long and 1 mm wide, with irregularly branches 0.8–17.0 × 0.1–1.0 mm, brown, becoming pale white toward the apex due to the formation of hymenium, fibrous, gradually attenuating toward the apex. ***Phialides*** emerging from the middle to upper regions of stromata, lageniform, broadly cylindrical, or swollen at base, hyaline, slightly guttulate, 6–11 × 3–8 (x̄ = 7 × 5, n = 20) μm, abruptly narrowing into a thin neck with slightly guttulate, 16–28 × 1–3 (x̄ = 22 × 2, n = 20) μm. ***Conidia*** 8–17 × 2–5 (x̄ = 13 × 3, n = 35) μm, narrowly cymbiform, clavate, and elongated fusiform, one-celled, hyaline, enveloped in a mucous sheath forming a globose head 3–8 (x̄ = 5, n = 15) μm in diameter. ***Teleomorph***: Undetermined.

**Figure 2.**
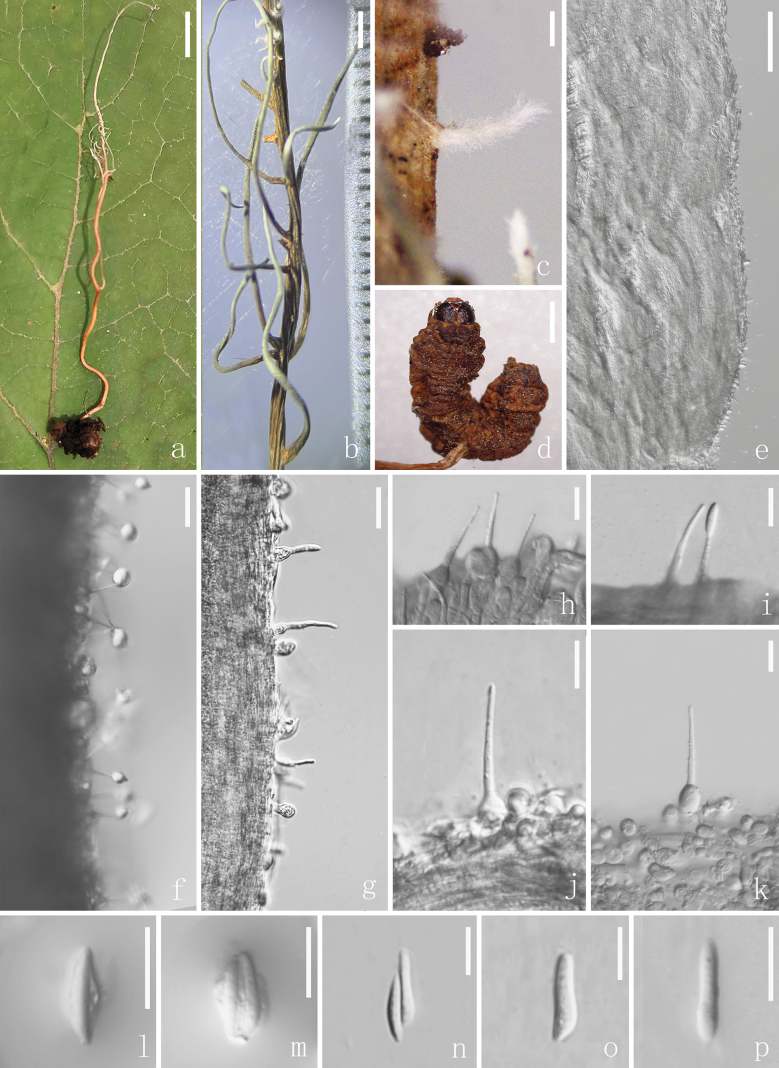
*Ophiocordycepstielingensis* (HKAS 135612, holotype) **a** stromata growing from the lepidopteran larva **b, c** close-up of branching stromata **d** close-up of host **e** enlargement of stromata **f** phialides with conidial mass **g–k** phialides **l, m** conidia limited in mucus sheath **n–p** conidia. Scale bars: 2 mm (**a, b**); 200 μm (**c**); 2 mm (**d**); 10 μm (**e, h–p**); 20 μm (**f–g**).

##### Material examined.

China • Liaoning Province, Tieling City, on a dead larva of Lepidoptera, Ting-Chi Wen, TL03 (HKAS 135612, ***holotype***).

##### Notes.

Multigene phylogenetic analysis showed that *O.tielingensis* forms a sister clade to *Hirsutellakuankuoshuiensis* with lower statistical values (76% ML / 0.95 PP) and grouped with *O.elongata* (anamorph: *Hirsutellagigantea*) (Sung et al. 2007a; Simmons et al. 2015b) (Fig. 1). All species share similarity in forming parasitic associations with larvae of Lepidoptera (Qu et al. 2018). *Ophiocordycepstielingensis* and *H.kuankuoshuiensis* were known only from their anamorphs. However, notable differences can be observed between *O.tielingensis* and *H.kuankuoshuiensis* in the morphologies of stromata, phialides, and conidia (Table 2). Hence, based on the biphasic approach, we confirm that our collection is qualified as a novel species of *Ophiocordycepss. str.*

**Table 2.** Morphological differences between *O.tielingensis* and *H.kuankuoshuiensis*.

Species	*O.tielingensis*	*H.kuankuoshuiensis*
Stromata (mm)	70, branched	86, unbranched
Phialides (μm)	Lageniform, broadly cylindrical, or swollen verrucose base, with a thin and verrucose neck, 16–28 × 1–3	Subulate or slender columnar base, with a long and narrow neck, 30–45 × 1–3
Conidia (μm)	8–17 × 2–5, narrowly cymbiform, clavate, and elongated fusiform, with a mucus	9.9–12.6 × 2.7–4.5, clavate, narrow fusiform, or botuliform, with a mucus
References	This study	Qu et al. 2018

#### ﻿ Ophiocordyceps keqinii

Taxon classificationFungiSordariomycetesOphiocordycipitaceae

S. W. Xie, T. C. Wen & D. P Wei sp. nov.

73EE18D4-3E96-5E93-A5CB-72A7E594F218

Index Fungorum: IF903219

Fig. 3

##### Etymology.

Named after an eminent Chinese mycologist, Prof. Ke-Qin Zhang, who has made a significant contribution to the studies of fungi in China.

**Figure 3.**
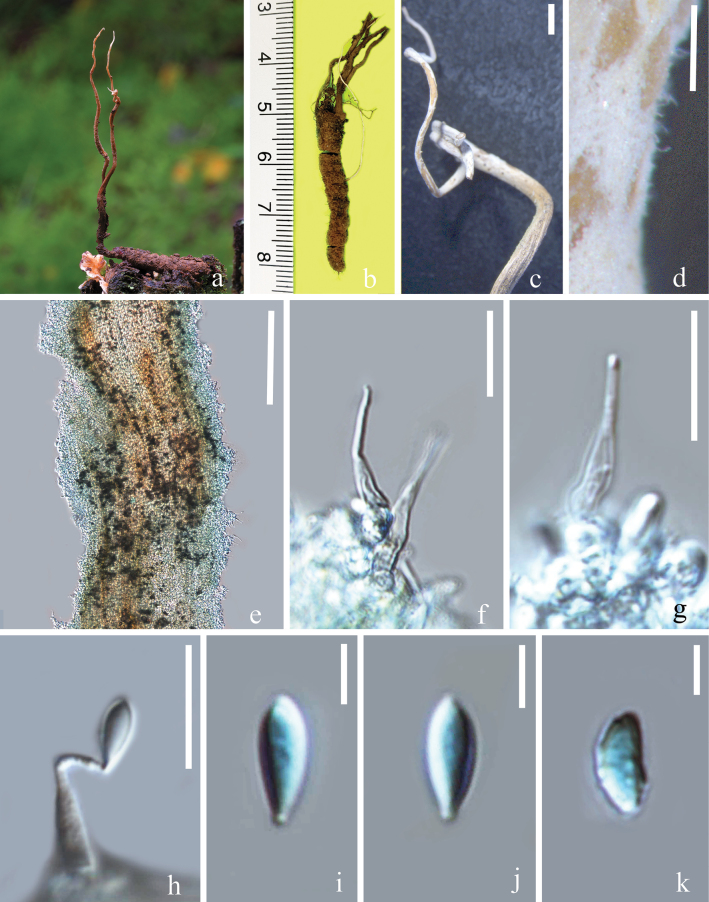
*Ophiocordycepskeqinii* (HKAS 135614, holotype) **a, b** stromata growing from the insect larva **c, d** close-up of stromata **e** stromata covered with hymenium **f–h** phialides **i–k** conidia. Scale bars: 200 μm (**c–e**); 15 μm (**f–h**); 5 μm (**i–k**).

##### Description.

***Anamorph*: *Stromata*** extending from the head of the lepidopteran larva, 15–90 × 0.3–1.1 mm, irregularly branched at upper part, cylindrical, fibrous, dark brown at base, becoming white toward the apex due to the formation of hymenium. ***Phialides*** exclusively formed at the apical region of stromata, hyaline, smooth-walled, cylindrical at the base 4–12 × 2–4 (x̄ = 7 × 3, n = 20) μm, narrowing rapidly to a long neck 6–16 × 0.7–2 (x̄ = 11 × 1, n = 20) μm. ***Conidia*** 3–12 × 2–5 (x̄ = 9 × 4, n = 20) μm, hyaline, semielliptical, ovoid with a round apex and obvious scars at base, one-celled, smooth-walled. ***Teleomorph***: Undetermined.

##### Material examined.

China • Yunnan Province, Honghe Prefecture, Amushan natural reserve, on a dead larva of Lepidoptera on the ground, Shi-Wen Xie, Y08 (HKAS 135614, ***holotype***).

##### Notes.

Phylogenetic analyses revealed that *O.keqinii* is sister to a clade comprising *O.macroacicularis* and *Hirsutellachangbeisanensis*, with strong statistical support (89% ML / 0.99 PP, Fig. 1). *Ophiocordycepsmacroacicularis* was found infecting lepidopteran larvae in Japan (Ban et al. 2015). According to the studies by Ban et al. (2015) and Zhou et al. (2015), they identified polyphialidic phialides in their strains of *O.macroacicularis*, which were absent in our collection. The comparison of nucleotide sequences showed that there are 17 bp differences (5 bp in ITS, 12 bp in *tef1-a*) between *O.keqinii* and *O.macroacicularis*, suggesting they are separate species.

*Hirsutellachangbeisanensis* was initially discovered on leafhoppers (Hemiptera) by Liang (1991) and restudied by Qu et al. (2017) based on a new collection occurring on Cicadellidae (Homoptera). *Hirsutellachangbeisanensis* is distinct from *O.keqinii* in having a verruculose neck, which is smooth-walled in our collection (Qu et al. 2017). Additionally, there are 23 bp differences in nucleotides (6 bp in ITS, 17 bp in *tef1-a*) between *O.keqinii* HKAS 135612 and *H.changbeisanensis* GZUIFR-hir160527, suggesting they are not conspecific. Hence, based on the differences in morphological characteristics (Table 3), multi-locus phylogenetic analyses, and base pair differences, we introduce *O.keqinii* as a new species of *Ophiocordyceps*.

**Table 3.** Differences in morphological characteristics of *Hirsutellachangbeisanensis*, *Ophiocordycepskeqinii*, and *O.macroacicularis*.

Species	*H.changbeisanensis*	*O.keqinii*	*O.macroacicularis*
Stromata (mm)	None	15–90 × 0.3–1.1, branched	97–166 × 1.3–2.4, branched
Phialides (μm)	Cylindrical base, 6.5–20.0 × 1.8–5.4, with a slender and verruculose neck 8.1–18.0	Cylindrical base 4–12 × 2–4, with a neck, 6–16 × 0.7–2	Awl-shaped, 21–63 long, 3–3.8 wide at base, 1.8–2.0 wide at neck
Conidia (μm)	Ellipsoid or orange-segment, 4.0–7.0 × 2.5–3.5, with a mucus	Semielliptical, ovoid with a round apex, 3–12 × 2–5	Orange-segment or oval, 8.1–10.8 × 2.7–5.4, with a mucus
References	Liang 1991; Qu et al. 2017	This study	Ban et al. 2015; Zhou et al. 2015

#### ﻿ Ophiocordyceps radiata

Taxon classificationFungiSordariomycetesOphiocordycipitaceae

(Petch) S. W. Xie, D. P Wei & T. C. Wen comb. nov.

86FB0130-9AB3-56C0-B6F8-53C9D7D5B67A

Index Fungorum: IF903448

Fig. 4

##### Basionym.

*Hirsutellaradiata* Petch, Trans. Br. Mycol. Soc. 19(3): 184 (1935) [1934].

**Figure 4.**
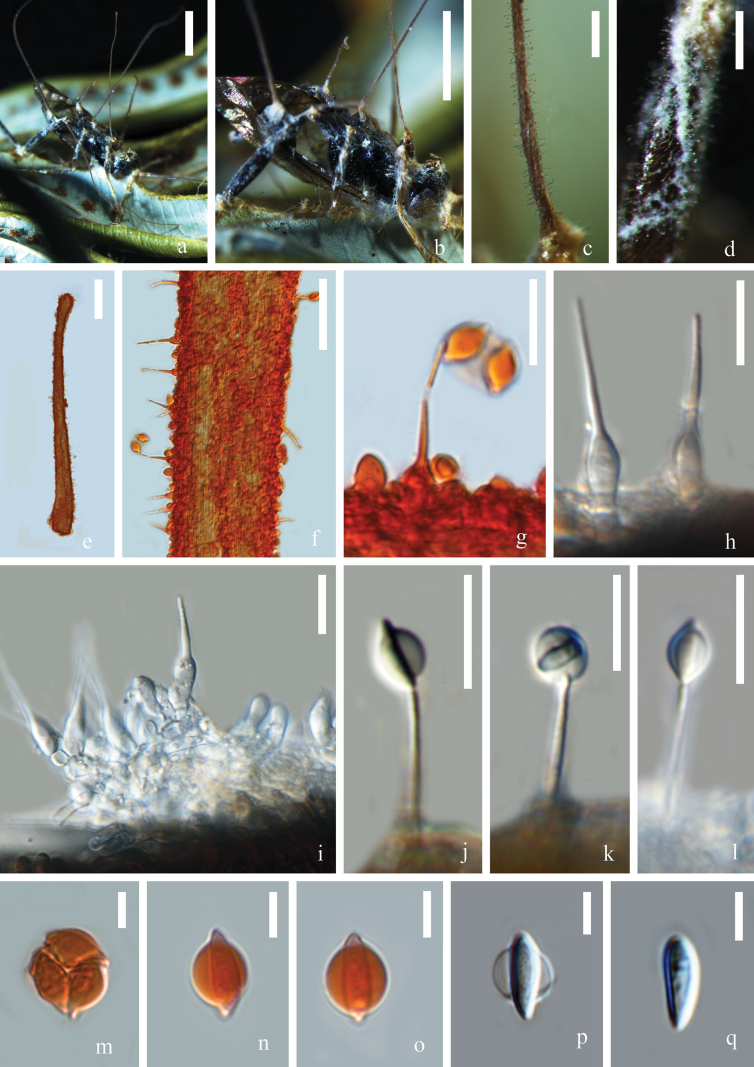
*Ophiocordycepsradiata***a, b** synnemata growing from the fly host **c** synnema-bearing conidiophores **d, i** sporodochia emerging from leg joints of host **e, f** synnema **g, j–l** conidia mass on tip of phialides **h** phialides **m** conidia mass **n–q** conidia. Scale bars: 2 mm (**a, b**); 200 μm (**c–e**); 50 μm (**f**); 15 μm (**g–l**); 5 μm (**m–q**).

##### Description.

***Anamorph*: *Synnemata*** up to 5.4 mm long, 0.04 mm wide, emerging from neck and leg joints of the host, multiple, unbranched, brown, filiform, slender, wiry, gradually attenuating toward the apex. ***Subiculum*** forming from leg joints of the host, white, composed of interlaced hyphae. ***Phialides*** laterally formed along synnemata or produced from subiculum, hyaline, aseptate, smooth-walled, cylindrical, 4–16 × 3–7 (x̄ = 10 × 4, n = 30) μm at the base, nrowing rapidly into a long neck 10–40 × 0.8–2 (x̄ = 19 × 1, n = 30) μm. ***Conidia*** 6–10 × 2–5 (x̄ = 9 × 3, n = 20) μm, hyaline, cymbiform, one-celled, smooth-walled, enveloped in a mucous sheath, forming a globose head 8–13 (x̄ = 10, n = 10) μm in diameter. ***Teleomorph***: Undetermined.

##### Material examined.

China • Yunnan Province, Honghe Prefecture, Amushan natural reserve, on fly (Diptera) attached to lower side of a living fern leaf, Shi-Wen Xie, TSQ13 (HKAS 135613).

##### Notes.

In the phylogenetic analyses, our new collection clustered with *Hirsutellaradiata* and *H.fusiformis*, forming a monophyletic clade with high statistical support (97% ML / 1 PP, Fig. 1). *Hirsutellaradiata* was initially found infecting a small fly attached to a leaf from Great Britain. It was characterized by filiform, brown, branched synnemata; phialides with conical to cylindrical bases and stout necks; cymbiform to oval conidia; and oval conidial masses (Petch 1935). *Hirsutellafusiformis* was introduced by Speare (1920) from a cricket in Hawaii. It has erect, straight, unbranched, nearly black synnemata; simple phialides with inflated basal portions tapering to a neck; and fusoid-cylindrical conidia. For the first time, Simmons et al. (2015b) used the DNA sequences of LSU, SSU, *tef1-a*, and *rpb1* gene regions of ‘*H.radiata*’ (from a specimen occurring on Diptera in Poland) and *H.fusiformis* (from a specimen occurring on *Brachyderesincanus* in the Netherlands) in their phylogenetic analyses. However, these sequences have not been linked to any morphological description, and epitypes were not designated. The close phylogenetic relationship between *H.radiata* and *H.fusiformis* was observed in this study and that of Simmons et al. (2015b), while it is undetermined whether they are conspecific. Morphologically, our specimen shares similarities with *H.radiata* in the association with a dipteran host, the filiform brown synnemata, and the cymbiform conidia; thus, we concluded our collection was *H.radiata*. According to our knowledge, this is the first geographical record of *H.radiata* in China. Besides, for the first time, we created the linkage between molecular data and the morphological characteristics of this species, thereby formally synonymizing *H.radiata* as *Ophiocordycepsradiata*.

## ﻿Discussion

### ﻿Systematics of *Ophiocordyceps* subclades with hirsutella-like anamorphs

Species with hirsutella-like anamorphs are distributed in most clades of *Ophiocordyceps*, with the exception of the *O.sphecocephala* clade (Fig. 1). The morphological characteristics of hirsutella-like phialides (including shape, size, branching patterns, and surface texture), along with their teleomorphs, exhibit significant variation across *Ophiocordyceps* clades (Table 4). Historically, Simmons et al. (2015b) established a foundational classification system for hirsutella-like anamorphs, delineating six subclades: *H.citriformis*, *H.guyana*, *H.nodulosa*, *H.sinensis*, *H.thompsonii*, and a distinct ‘ant pathogen’ subclade. Qu et al. (2018) subsequently provided comprehensive morphological descriptions for the first five subclades. Notably, Araújo et al. (2018) redefined the “ant pathogen” subclade as the *O.unilateralis* clade. Building on these frameworks, recent studies have expanded the phylogenetic scope by proposing additional hirsutella-like clades, such as the *O.sobolifera* and *O.ravenelii* clades (Wang et al. 2018; Fei et al. 2024; Sun et al. 2024). However, taxonomic inconsistencies persist: Dai et al. (2024) merged four subclades (*H.guyana*, *H.nodulosa*, *H.sinensis*, and *H.thompsonii*) into a broader *O.sinensis* clade. Mongkolsamrit et al. (2024) incorporated four hirsutella-linked clades (*O.blattae*, *O.elongata*, *O.ravenelii*, and *O.sobolifera*) in their analysis, revealing partial overlap between groups (e.g., *O.blattae* with *H.citriformis*; *O.elongata* with *H.thompsoni*i). These conflicting nomenclature systems across studies highlight the taxonomic complexity of *Ophiocordyceps* subclades. Critically, the morphological diversity of hirsutella-like anamorphs remains systematically unclear, obscuring potential correlations between anamorphs, teleomorphs, and host-specific adaptations. This study reassessed the subclades of *Ophiocordyceps* with hirsutelloid anamorphs and proposed two novel clades (*O.issidarum* and *O.acicularis*), which have not been recognized in prior taxonomic classifications.

**Table 4.** The synopsis of the phylogenetic lineage of hirsutella-like anamorphs in *Ophiocordycepss. str.*

Hirsutella-like subclade	Description
*O.sinensis* clade	This clade comprises taxa characterized by phialides with cylindrical, slender, or subulate bases that gradually taper into a warted neck (Simmons et al. 2015b; Qu et al. 2018). The teleomorphs of this clade produce superficial perithecia and filiform, multiseptate, whole ascospores (Dai et al. 2024).
*O.issidarum* clade	This clade shares large phialides with a cylindrical basal portion (Qu et al. 2018). The teleomorphs of this clade have been known from *O.issidarum* (Hyde et al. 2017) and *O.spataforae* (Luangsa-ard et al. 2018). Both of them produce superficial perithecia and filiform, multiseptate, whole ascospores (Hyde et al. 2017; Luangsa-ard et al. 2018).
*O.acicularis* clade	This clade is composed of many cryptic species occurring on lepidopteran larvae, except for *H.leigongshanensis*, which infects coleopteran larvae (Tasanathai et al. 2020). The anamorphs of this clade are characterized by the helical neck of the phialides (Mains 1950; Liang 1990a, 1990b). The teleomorphs of this clade produce superficial perithecia and needle-like or filiform, whole ascospores (Tasanathai et al. 2020).
*O.blattae* clade	The members of this clade are specialized parasites on Blattodea (cockroaches and termites) and produce superficial or immersed perithecia and filiform, multiseptate, whole ascospores (Araújo et al. 2021). The anamorphs in this clade are produced at the terminal part of the stromata (Tasanathai et al. 2019). Its phialides are inflated at the base, and the conidia are globose or fusiform with a warty surface or mucous sheath (Qu et al. 2018, Tasanathai et al. 2019, 2022).
*O.elongata* clade	The teleomorphs of this clade have been known from *O.alboperitheciata*, *O.elongata*, *O.capilliformis*, and *O.xifengensis* (Fei et al. 2024; Mongkolsamrit et al. 2024). This clade contains species pathogenic to a variety of insect taxa and produces terminal or intercalary fertile parts, immersed or superficial perithecia, and narrowly fusiform, whole ascospores (Fei et al. 2024; Mongkolsamrit et al. 2024). The anamorphs of this clade are unique in producing many branches along the stromata, and the conidia usually are encompassed by a mucous sheath (Tan et al. 2023; Mongkolsamrit et al. 2024).
*O.unilateralis* clade	This clade consists of the *O.unilateralis* core clade and *O.kniphoﬁoides* subclade. These two groups are different in the ascomata morphologies (Araújo et al. 2018). Species in the *O.unilateralis* core clade produce brown to black ascomata laterally attached to stromata, while species in the *O.kniphoﬁoides* subclade produce orange ascomata covering 360° of the stalk (Araújo et al. 2018). Phialides in this clade generally are monophialidic and produce limoniform conidia at the tip. Some species of this clade produce polymorphic phialides, which are defined as Hirsutella A-type, Hirsutella B-type, and Hirsutella C-type (Evans and Samson 1982; Evans and Samson 1984; Evans et al. 2011; Araújo et al. 2018).
*O.sobolifera* clade	This clade encompasses fungi pathogenic to cicada and coleopteran larvae (Zou 2022; Mongkolsamrit et al. 2024), sharing morphological traits in producing cylindrical ascomata at the subterminal part of stromata ended with fertile tips, immersed perithecia, multiseptate, disarticulating ascospores. The anamorphs in this clade are nomo- to polyphialidic phialides with a nearly globose base abruptly narrowing into a hair-like, long neck terminated in subglobose conidia without an evident mucous sheath (Lao et al. 2021; Zou 2022; Mongkolsamrit et al. 2024).
*O.ravenelii* clade	This clade comprises the taxa that prefer larvae of Coleoptera and share morphology in producing yellow, orange, or brown stromata, forming immersed perithecia on terminal or lateral fertile parts, and filiform, multiseptate ascospores fragmenting into cylindrical secondary ascospores at maturity (Wang et al. 2015; Mongkolsamrit et al. 2024). The mucous sheaths are commonly absent in species of this clade (Wang et al. 2015).

**Figure 5.**
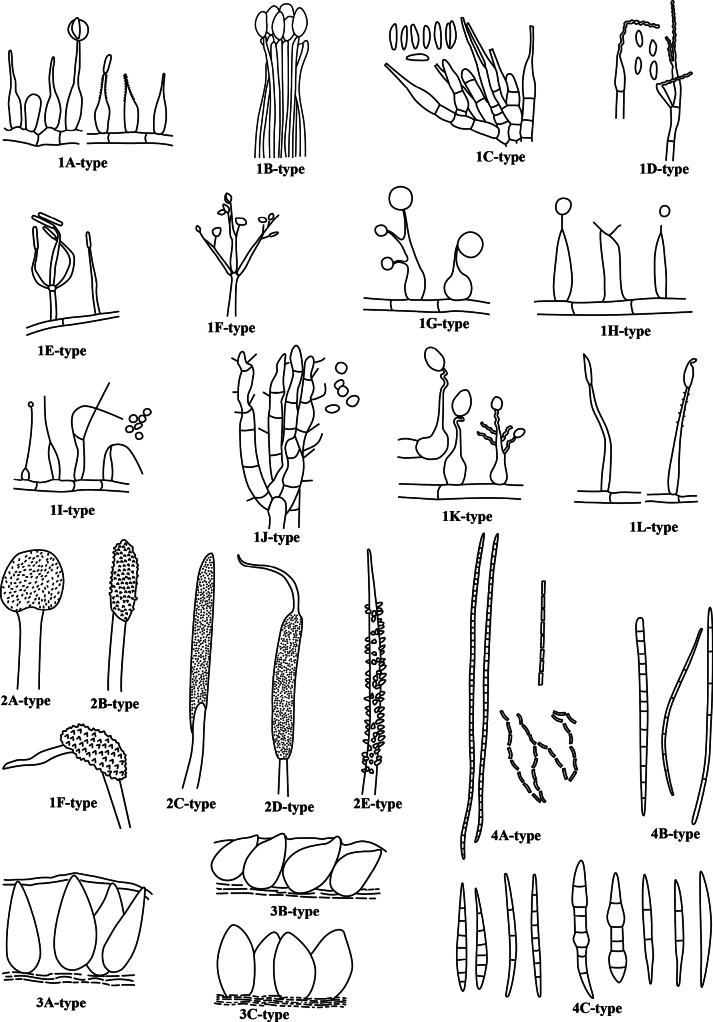
The anamprphs and teleomorphs characteristics of *Ophiocordyceps* species with hirsutella-like anamorph **1A–1L type** hirsutella-like types **2A–2F type** stromatal types **3A–3C** perithecial arrangements **4A–4C type** ascospore shapes **2A-type**, terminal **2B, 2C type** subterminal **2D, 2E type** intercalary **2F-type** lateral **3A-type** immersed **3B-type** obliquely immersed **3C-type** superficial **4A-type** filiform, multiseptate, disarticulating **4B-type** needle-like or filiform, whole **4C-type** vermiform.

Furthermore, we have prepared a checklist of *Ophiocordyceps* species with hirsutella-like anamorphs (see the Suppl. material 1). There are 95 species of *Ophiocordyceps* that have been reported to produce hirsutella-like anamorphs that are various in shape, branching, ornamentation, and arrangement of phialides, as shown in Fig. 5.

#### ﻿1A-type phialide

The 1A-type phialide corresponds to *Hirsutella* Type A as described by Araújo et al. (2018). It is characterized by monophialidic, cylindrical at the base abruptly narrowing into a thin neck, and it is commonly found in *O.unilateralis* clade. Teleomorphs of this clade are featured with lateral fertile cushions, immersed perithecia, and whole ascospores (Araújo et al. 2018; Tang et al. 2023b, 2023c). The 1A-type phialides usually co-occur with the teleomorph of *O.unilateralis* clade, and they are associated with apical region of stromata (Wei et al. 2022).

#### ﻿1B-type phialide

The 1B-type phialide corresponds to *Hirsutella* Type B as described by Evans and Samson (1982). It is cylindrical, finely echinulate, and accumulated at terminal regions and only found from *O.camponoti-novogranadensis* (Evans and Samson 1982; Evans et al. 2011). Phialides develop acrogenously at the synnematal apex, with their supporting synnemata arising from joint or foot of all legs. Synnemata upright, black, cylindrical at the base, tapering towards apex and broadening into a globose head.

#### ﻿1C-type phialide

The 1C-type phialide is unique by its intergraded, septate conidiophores terminating in flask-shaped phialides, which can be seen in the Hirsutella C-type of *O.unilateralis* complex (Evans and Samson 1984; Kobmoo et al. 2015; Wei et al. 2022). *Hirsutella* C-type phialides are produced from brown cushions (sporodochia) on the leg and antennal joints of ants.

#### ﻿1D-type phialide

The 1D-type phialide is unique with an undulate neck, which is only found in *H.dendritica*, a species without molecular data (Samson and Evans 1991).

#### ﻿1E-type phialide

The 1E-type phialide is curved and gradually attenuated toward the apex from the middle part, and the conidia are cylindrical. This type of hirsutella has been linked to *O.formosana*, which has a terminal fertile part, obliquely immersed perithecia, and filiform ascospores fragmenting into cylindrical and truncated part-spores (Li et al. 2005; Wang et al. 2015).

#### ﻿1F-type phialide

The 1F-type phialide is branching and becoming thread-like at the subterminal region. This type of phialide has been found from culture of *O.kobayasii*, with its anamorphs being defined as hymenostilbe-like on the natural specimen and as acremonium-like in artificial culture (Thanakitpipattana et al. 2020). However, we recognize both of the mentioned anamorphs as hirsutella-like following the line drawing provided by Mongkolsamrit et al. (2023). Additionally, the significant morphological difference of anamorphs on natural specimens and in culture suggests that the substrate can shape anamorphic traits. Thus, phialide morphology from different substrates is incomparable for species delimitation.

#### ﻿1G-type phialide

The 1G-type phialide is characterized by the globose base and short neck terminating in a single globose conidium. This was observed from cultures of *Hirsutellaminnesotensis*, a species pathogenic to nematodes (Chen et al. 2000).

#### ﻿1H-type phialide

The 1H-type phialide presents a flask shape with the base tapering towards a short, thread-like neck. This type of phialide has been reported from *O.spataforae* (Luangsa-ard et al. 2018), *O.geometridicola* (Luangsa-ard et al. 2018), *O.flavida* (Mongkolsamrit et al. 2021), and *O.ovatospora* (Tang et al. 2022).

#### ﻿1I-type phialide

The 1I-type phialide is mono- to polyphialidic and can be recognized by its inflated base and filiform, long neck-producing globose conidia. Mongkolsamrit et al. (2024) have described *O.ratchaburiensis*, *O.brunnea*, and *O.kohchangensis*, with the 1I-type phialide being observed from cultures. The three mentioned species are featured with intercalary fertile parts ending with sterile tips, immersed perithecia, filiform, disarticulating ascospores, and occurrences on coleopteran larvae.

#### ﻿1J-type phialide

The 1J-type phialide is polyphialidic with cylindrical, multiseptate base and short, thread-like necks and often intergraded into a hymenial layer. This type of phialide was only found on natural specimens of *O.ratchaburiensis* and *O.naomipierceae* (Araújo et al. 2018; Mongkolsamrit et al. 2024).

#### ﻿1K-type phialide

The distinctiveness of 1K-type phialides is the inflated base narrowing into one to several thin necks apically twisted in a characteristic helix. It is worth mentioning that the co-occurrence of twisted neck and smooth neck can be observed in one species such as *O.pseudoacicularis*, *O.longistromata*, and *O.retorta* (Luangsa-ard et al. 2018; Qu et al. 2018; Tasanathai et al. 2020), indicating that the ornamentation of the neck is not significant for interspecific demarcation. The 1K-type phialides commonly are observed from cultures isolated from Lepidoptera-pathogenic species with intercalary fertile parts, superficial perithecia, and needle-like, filiform, whole ascospores.

#### ﻿1L-type phialide

1L-type phialides are slenderer than 1A-type phialides, narrowing gradually into a neck with warty protrusions and often coming with conidia enveloped in a mucous sheath. This type of phialide often is found on the culture of Lepidoptera-pathogenic species with intercalary fertile parts, immersed or superficial perithecia, and whole ascospores (Ban et al. 2015; Luangsa-ard et al. 2018).

### ﻿Morphological diversity of teleomorphs with hirsutella-like anamorphs

**Figure 6.**
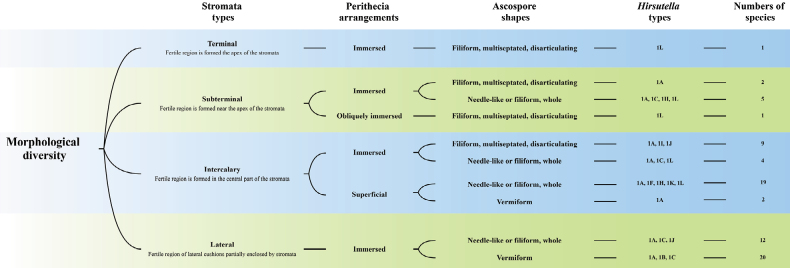
The relationship of anamorphs and teleomorphs’ characteristic state of the Suppl. material 1.

The morphological diversity of teleomorphs linked with hirsutella-like anamorph is based on the Suppl. material 1 and presented in Fig. 6. It is shown that terminal, subterminal, intercalary, and lateral stromata types are linked with hirsutella-like anamorphs. Teleomorphs with lateral stromata type, immersed perithecia, and whole ascospores often come with 1A, 1B, and 1C-type phialides. This combination of teleomorphs and anamorphs has been found in up to 20 species. Significantly, 1L-type phialides are often found from cultures of the *O.unilateralis* complex. Up to 19 species have been described to have an intercalary stromatal type, superficial perithecia, needle-like or filiform whole ascospores, and 1A, 1F, 1H, 1K, and 1L type phialides. We categorized these teleomorph-anamorph combinations into several different groups, which provides the guideline for species identification for future work.

## Supplementary Material

XML Treatment for Ophiocordyceps tielingensis

XML Treatment for Ophiocordyceps keqinii

XML Treatment for Ophiocordyceps radiata

## ﻿Additional information

### Author ORCIDs

Shi-Wen Xie https://orcid.org/0009-0005-2803-0302

De-Ping Wei https://orcid.org/0000-0002-6576-2239

Xing-Can Peng https://orcid.org/0000-0002-7271-7639

Ji-Chuan Kang https://orcid.org/0000-0002-6294-5793

Zhang-Jiang He https://orcid.org/0000-0002-7120-1227

Shi-Ke Huang https://orcid.org/0000-0002-2936-396X

Xian Zhang https://orcid.org/0009-0008-0919-4303

Zhong-Liang Liu https://orcid.org/0009-0007-9519-1418

Jing Bu https://orcid.org/0009-0006-6861-7770

Nalin N. Wijayawardene https://orcid.org/0000-0003-0522-5498

Ting-Chi Wen https://orcid.org/0000-0003-1744-5869

### Data availability

All of the data that support the findings of this study are available in the main text or Supplementary Information.

## Supplementary materials

Supplementary material 1 The relationship of anamorph and teleomorph characteristics state. Data type xlsx https://binary.pensoft.net/file/1364232This dataset is made available under the Open Database License (http://opendatacommons.org/licenses/odbl/1.0/). The Open Database License (ODbL) is a license agreement intended to allow users to freely share, modify, and use this Dataset while maintaining this same freedom for others, provided that the original source and author(s) are credited.Shi-Wen Xie, De-Ping Wei, Jun-Zhi Qiu, Xing-Can Peng, Ji-Chuan Kang, Zhang-Jiang He, Zeng-Zhi Li, Chun-Ru Li, Shi-Ke Huang, Xian Zhang, Zhong-Liang Liu, Jing Bu, Nalin N. Wijayawardene, Ting-Chi Wen
